# The safety of spinal manipulative therapy in children under 10 years: a rapid review

**DOI:** 10.1186/s12998-020-0299-y

**Published:** 2020-02-25

**Authors:** Melissa Corso, Carol Cancelliere, Silvano Mior, Anne Taylor-Vaisey, Pierre Côté

**Affiliations:** 1 Faculty of Health Sciences, Centre for Disability Prevention and Rehabilitation, Ontario Tech University and CMCC, 2000 Simcoe St N, Oshawa, ON L1G 0C5 Canada; 2 Canadian Memorial Chiropractic College, 6100 Leslie St, Toronto, ON M2H 3J1 Canada

**Keywords:** Adverse event, Child, Pediatric, Safety, Spinal manipulation, Spinal mobilization

## Abstract

**Introduction:**

The safety of spinal manipulative therapy (SMT) in children is controversial. We were mandated by the College of Chiropractors of British Columbia to review the evidence on this issue.

**Objectives:**

We conducted a rapid review of the safety of SMT in children (< 10 years). We aimed to: 1) describe adverse events; 2) report the incidence of adverse events; and 3) determine whether SMT increases the risk of adverse events compared to other interventions.

**Evidence review:**

We searched MEDLINE, CINAHL, and Index to Chiropractic Literature from January 1, 1990 to August 1, 2019. We used rapid review methodology recommended by the World Health Organization. Eligible studies (case reports/series, cohort studies and randomized controlled trials) were critically appraised. Studies of high and acceptable methodological quality were included. The lead author extracted data. Data extraction was independently validated by a second reviewer. We conducted a qualitative synthesis of the evidence.

**Findings:**

Most adverse events are mild (e.g., increased crying, soreness). One case report describes a severe adverse event (rib fracture in a 21-day-old) and another an indirect harm in a 4-month-old. The incidence of mild adverse events ranges from 0.3% (95% CI: 0.06, 1.82) to 22.22% (95% CI: 6.32, 54.74). Whether SMT increases the risk of adverse events in children is unknown.

**Conclusion:**

The risk of moderate and severe adverse events is unknown in children treated with SMT. It is unclear whether SMT increases the risk of adverse events in children < 10 years.

## Introduction

The treatment of children with spinal manipulative therapy (SMT) by chiropractors is controversial [1–3]. A recent study of Ontario chiropractors suggests that 5.5% of patients who consulted a chiropractor in the previous month are between the ages of 0 and 14 years [4]. A cross-sectional survey of 140 chiropractors in Alberta indicates that all respondents treated children between 0 and 18 years of age and 13% of all visits in the previous month were with children [5]. Worldwide, the estimated 12 month median utilization and interquartile range (IQR) of chiropractic care is 8.1% (IQR 3.8–20.00), with a lifetime median utilization of 11.1% (IQR 4.0–21.6) for patients 18 years old or less [6]. Children visit chiropractors for a variety of reasons, including health promotion, asthma, otitis media, allergies, infantile colic, tonsillitis, ADHD, and enuresis, but most commonly for musculoskeletal (MSK) problems [6–12].

Although the effectiveness of chiropractic care for the management of pediatric MSK and non-MSK conditions is debatable, most of the controversy surrounds the safety of SMT in children [3, 13]. A survey of Canadian pediatricians suggests that serious adverse events of SMT in children may be rare [14]. Previous systematic reviews identified case reports of serious adverse events of SMT including death and temporary paraplegia [13, 15]. However, these systematic reviews did not assess the methodological quality of the included studies. This is problematic because of the high risk of bias associated with such reports [16, 17]. Although previous systematic reviews reported on the type and frequency of adverse events, they did not describe the incidence of adverse events, or determine whether SMT increases the risk of adverse events compared to other interventions [13, 15, 18]. Therefore, little evidence is available to understand the risk of adverse events associated with SMT in children [13, 15, 18]. There is a need to update these reviews to inform the current policy debate about the safety of SMT in children.

To assist in informing this public debate, the College of Chiropractors of British Columbia called for a review of the evidence on the safety of SMT for children under the age of 10 years [19]. At the request of the College of Chiropractors of British Columbia, we conducted an independent rapid review of observational studies and randomized controlled trials (RCTs) to investigate the type and risk of adverse events in children under the age of 10 years (0–9 years) who receive SMT from any health care provider. Specifically, we aimed to: 1) describe the reported adverse events; 2) report the incidence of adverse events; 3) determine whether SMT is associated with an increased risk in adverse events compared to other interventions used to manage children for any health condition, or the promotion of health and wellness.

## Methods

Rapid reviews are used by health decision-makers (clinicians, patients, managers, and policy makers) who need timely access to health information to plan, develop and implement health policies [20, 21]. Rapid reviews are a valuable method to provide actionable and relevant evidence to make informed decisions in a short amount of time [20, 21]. They follow the key principles of knowledge synthesis used in systematic reviews, including clear objectives, a priori definition of eligibility criteria, a systematic search for relevant evidence, assessment of validity of findings and a systematic presentation and synthesis of results [20]. However, certain components of the systematic review process are simplified or narrowed to produce information in a timely manner, such as a focused research question, limited databases searched, and one reviewer for screening, critical appraisal and data extraction using standardized established procedures [21]. We followed the methodology recommended by the World Health Organization [20].

### Protocol and registration

We reported our review according to the PRISMA and PRISMA harms checklists (Additional file 3) [22, 23]. We registered our review with the International Prospective Register of Systematic Reviews (PROSPERO) on August 1, 2019 (CRD42019145581).

### Eligibility criteria

#### Participants

We included studies of children between 0 and 9 years of age [24] who received spinal manipulation or mobilization for the prevention or management of any health condition (i.e., MSK or non-MSK disorders) or for the promotion of health and wellness. We did not restrict our review to studies that reported adverse events, rather we considered any study that focused on the treatment of children between 0 and 9 years old with SMT.

#### Interventions

SMT includes spinal manipulation and spinal mobilization provided by any type of provider. Spinal manipulation includes techniques incorporating a high-velocity, low-amplitude impulse or thrust applied at or near the end of a joint’s passive range of motion [25, 26]. Spinal mobilization includes techniques incorporating a low-velocity and varying amplitude oscillatory movement within a joint’s passive range of motion [26–28]. Spinal manipulation and mobilization involve manual and mechanically-assisted procedures.

#### Comparators

We considered all control interventions tested in cohort studies and RCTs to determine the relative risk of adverse events. This may include, but are not limited to placebo, sham manual therapies, wait listing, usual care, no interventions, medication and other manual therapies.

#### Outcomes

We investigated adverse events including indirect harms (Table 1). We defined adverse events as any unfavorable sign, symptom, or disease temporally associated with the treatment, whether or not caused by the treatment [29]. We used predefined categories to rate them as mild, moderate, severe or serious [29]. We also considered indirect harms, where the use of an intervention delays a diagnosis or treatment, and such delay holds a potential harm [14]. We used the classification adapted by a multi-disciplinary team of content experts and providers of SMT [29]. We critically appraised studies reporting on adverse events and then classified the severity and nature of all reported adverse events according to the definitions provided in Table 1. To be eligible for inclusion, case reports and case series had to describe that the presence (or absence) of adverse events was investigated.

**Table 1:** Classification of adverse events [14, 29]

Mild	Asymptomatic or mild symptoms, requiring self-care only to alleviate symptoms (e.g. ice/heat, over-the-counter analgesic).
Moderate	Limiting age-appropriate activities of daily living (e.g. work, school) OR sought care from a medical doctor.
Severe	Medically significant but not immediately life-threatening; temporarily limits self-care (e.g. bathing, dressing, eating); OR urgent or emergency room assessment sought.
Serious	Results in death OR a life-threatening adverse event OR an adverse event resulting in inpatient hospitalization or prolongation of existing hospitalization for more than 24 h; a persistent or significant incapacity or substantial disruption of the ability to conduct normal life functions.
Indirect harms	The use of intervention may cause a delay in diagnosis or treatment and the delay itself carries the potential harm.

#### Study designs

Eligible study designs included: case report, case series, case-control study, cohort study or RCT. We used case reports, case series, case-control studies, cohort studies and RCTs to describe the adverse events reported in the literature (aim 1). We used cohort studies and RCTs to determine the incidence of adverse events associated with SMT (aim 2), and to determine the relative risk of adverse events associated with SMT compared to other interventions (aim 3).

We excluded guidelines, letters, editorials, commentaries, unpublished manuscripts, dissertations, government reports, books and book chapters, conference proceedings, meeting abstracts, lectures and addresses, consensus development statements, guideline statements, cadaveric, laboratory or animal studies, qualitative studies, systematic reviews and meta-analyses.

### Information sources

We developed our search strategy in consultation with a health sciences librarian, and a second librarian reviewed the strategy to ensure accuracy. We systematically searched three databases that thoroughly index the manual therapy literature published by various health professions from January 1, 1990 to August 1, 2019: MEDLINE (U.S. National Library of Medicine, through Ovid Technologies Inc.), Cumulative Index to Nursing and Allied Health (CINAHL, through EBSCO*host*), and Index to Chiropractic Literature (ICL, Chiropractic Library Collaboration). Search terms consisted of subject headings specific to each database (e.g., MeSH in MEDLINE) and free text words relevant to our objectives and study design [see Additional file 1]. We restricted our search to papers published in English.

### Study selection

We used a two-phase screening process to identify eligible studies. In phase one screening, we reviewed titles and abstracts and classified articles as relevant, possibly relevant or irrelevant. During phase two screening, we reviewed the full text of possibly relevant articles for final determination of eligibility.

A trained investigator (MC) conducted all of the screening. Prior to phase one screening, we validated the quality of screening by MC. Ten percent of all eligible articles were randomly selected and the titles and abstracts of these articles were screened independently by a second experienced investigator (PC). A 95% level of agreement was required between two reviewers before moving to full phase one screening. Once the 95% agreement was achieved, one reviewer (MC) completed phase one and two screening.

### Risk of Bias in individual studies

The lead author (MC) critically appraised the internal validity of relevant articles using the Scottish Intercollegiate Guidelines Network (SIGN) criteria for RCTs, cohort studies and case-control studies [30, 31]. The SIGN methodology provides the reviewer with a list of standardized criteria to determine the risk of bias related to selection bias, measurement bias and confounding. When evaluating an RCT, the reviewer assessed methods of randomization, concealment, blinding, comparability of baseline characteristics, contamination, outcome measurement, loss to follow-up, intention-to-treat and between site differences (for multi-center RCTs). In cohort studies, the items focus on the source population, participation rate, drop-out rate, outcome measurement, blinding, exposure measurement, confounders and statistical analysis. We did not identify any case-control studies; thus, we do not describe the SIGN criteria for this study type.

There are no SIGN criteria for case reports or case studies. Therefore, we adapted the critical appraisal tool proposed by Murad et al. to assess the quality of case reports and case series. We modified the tool by creating a series of critical appraisal criteria and notes similar to the SIGN criteria [see Additional file 2] [17]. The adapted Murad tool allowed us to critically appraise patient selection, exposure and outcome measurement, alternative causes, challenge-rechallenge phenomenon, dose-response, and length of follow-up.

We also included a quality control step in the critical appraisal of studies. The investigator who assessed the risk of bias of the studies (MC) presented a summary of the critically appraised papers to three experienced methodologists (PC, SM, CC) who validated the outcome of the appraisals. Disagreements regarding the internal validity of papers were resolved through discussion. We restricted our synthesis to studies agreed judged to have a low risk of bias. The lead author created risk of bias tables for all eligible studies (including low and high risk of bias studies), which were validated by the other investigators (PC, SM, CC). Studies were rated as high quality, acceptable, low quality or unacceptable.

### Data items

Information extracted from each RCT included participant characteristics (age, indications for treatment or condition treated, and location); sample size; type and description of intervention; type and description of comparison group; follow-up period; method of outcome ascertainment; and number and description of adverse events. Information extracted from each cohort study included source population; sample size; participant characteristics (age, indications for treatment or condition treated); exposure/description of intervention; method of outcome ascertainment; confounders; follow-up period; and number and description of adverse events. Information extracted from case reports and case series included participant characteristics (age, indications for treatment or condition treated); type and description of intervention; method of outcome ascertainment; follow-up period; and number and description of adverse events.

### Data extraction

The lead author (MC) extracted data from high and acceptable quality studies and built evidence tables stratified by research objective (Tables 2, 3, 4 and 5). Data extraction was validated by one of three reviewers (PC, CC, SM). We contacted the study authors when clarification or additional information/data was necessary to build the evidence tables [32, 33]. Evidence tables summarized the information relevant to each objective and we used this information to create the summary statements. We used the terminology used by authors to describe adverse events when building our evidence table. For example, authors may have labelled adverse events as “side effects” or adverse reactions [34, 35]. We considered these terms were synonymous to adverse events in our synthesis.

**Table 2:** Evidence Table: Randomized Controlled Trials^a^

Randomized Controlled Trials^a^						
Author(s), Year	Subjects & Setting; n	Interventions; n	Comparisons; n	Follow-up	Outcomes	Key Findings Description Incidence: %, (95% CI)
Miller et al., 2012 [61]	Infants (< 8 wks), born at gestational age > 37 wks, birth weight > 2500 g, no other conditions or illness, unexplained persistent crying, Anglo-European College of Chiropractic; *n* = 104	SMT w/o blinding; *n* = 33 SMT w/ blinding; *n* = 35 SMT: low force tactile pressure to spinal joints and paraspinal muscles (estimated at 2 N of force without rotation of the spine), where dysfunction was noted on palpation, pragmatic, individualized to exam findings of the individual; chiropractic manual therapy of the spine	No SMT w/ blinding; *n* = 34	10 days or complete resolution of symptoms (discharged) SMT w/o blinding: 10 day, *n* = 26; discharged, *n* = 7 SMT w/ blinding: 10 day, *n* = 30; discharged, *n* = 5 No tx w/ blinding: 10 day, *n* = 22; dropped out, *n* = 12	Parent report of any AE during tx period	One child in comparison group reported an AE of increased crying. Incidence of increased crying in comparison group: 2.94% (0.52, 14.92)
Sawyer et al., 1999 [32]	6 mos – 6 years, > 3 episodes acute otitis media in last year, middle ear effusion at first 2 visits; Northwestern College of Chiropractic, Bloomington, Minnesota; *n* = 20	10 tx over 4 weeks; *n* = 9 Full spine HVLA SMT, emphasis on upper cervical area	10 placebo over 4 weeks; *n* = 11 Manual static and motion palpation and light touch of specific spinal segments. Identical to active tx without HVLA.	Immediately after completion of tx, 1 month after completion of tx	Direct verbal inquiry at each visit	One parent in tx group reported their child had mid-back soreness after 1 tx which resolved after a few days. Another child was reported to be irritable for a short time after tx. Incidence of AE in the tx group: 22.22% (6.32, 54.74) One parent of a child in the placebo group reported excessive crying after tx. Incidence of AE in the placebo group: 9.09% (1.62, 37.74) RR: 2.44 (0.26, 22.8)

*AE* Adverse events, *D/t* Due to, *FU* Follow-up, *HA* Headache, *HVLA* high velocity low amplitude, *SMT* spinal manipulative therapy, *Months* mos, *RR* relative risk, *Tx* Treatment, *W/o* Without, *W/* With, *Wks* Weeks

^a^Data reported in this table only relates to adverse events, not benefits of treatment

**Table 3:** Evidence Table: Cohort Studies^a^

Author(s), Year	Source Population	Sample Characteristics	Exposure	Outcomes	Confounders	Key Findings
Saedt et al., 2018 [33]	Infants < 27 wks, referred w/ indications of upper cervical dysfunction, w/o causative concomitant pathology, potential underlying pathology, &/or red flags; Netherlands	Mean age: 11.2 wks62.3% male Reasons for seeking care: clear positional preference, restlessness and abnormal head position; *n* = 307	Mild mobilization techniques focusing on atlas (C1) in relation to C0-C2. Average impulse of 11–20 N.	Harms recorded by manual therapists via questionnaire and physical exam post-exam: Mild: transient side effect lasting < 24 h Moderate: requiring medical and/or general practitioner tx Severe: requiring hospital tx, AE	N/A	Severe: 0% Moderate: 0% Mild: Vegetative responses after mobilization were reported: - Flushing: 17.8% (14.03, 22.59) - Hyper-extension: 4.3% (2.49, 7.11) - Perspiration: 3.6% (2.01, 6.30) - Gastro-esophageal reflux: 0.3% (0.06, 1.82) Short breathing pattern changes: 9.2% (6.39, 12.87)

*AE* Adverse events, *D/t* Due to, *FU* Follow-up, *HA* Headache, *HVLA* high velocity low amplitude, *SMT* spinal manipulative therapy, *Months* mos, *N/A* not applicable, *RR* relative risk, *Tx* Treatment, *W/o* Without, *W/* With, *Wks* Weeks

^a^Data reported in this table only relates to adverse events, not benefits of treatment

**Table 4:** Evidence Table: Case Series

Author(s), Year	Subjects & Setting; n	Intervention(s)	Method of Measurement of AE	Follow-up	Key Findings^a^
Iyer, 2017 [34]	Patient A: 7-month-old; male; difficulty with constipation since birth; Patient B: 7-month-old; male; constipation since birth; *n* = 2	Gentle acupressure stimulation on feet, scar tissue mobilization, gentle manipulation was applied to the cervical and lumbar segments and SI joints, with the line of drive being posterior to anterior and lateral to medial (magnitude of thrust and force adapted to patient age and neuromusculoskeletal maturity); patient A also had DNS rehabilitation Patient A: 2x/week, 5 wks Patient B: 2x/week, 4 wks	Cannot say	During course of tx	No adverse reactions were reported to occur with the intervention
Young, 2017 [56]	Patient A: 26-month-old; female; crying on waking complaining of neck pain; no known previous accidents or injuries during play, played in bouncy house, no complaints of pain the day of; pain increased on 4th day with reduced range of motion and torticollis; Patient B: 33-month-old; male; playing in bouncy house, no complaint of pain or injury, awoke next morning with right-sided neck pain, 1 day later could not turn head to the right; *n* = 2	Activator 4 applied at its lowest force setting, ischemic compression to trigger points to patient tolerance, home since and range of motion exercises Patient A: 1 tx Patient B: 4 tx over 2 weeks, with 3 tx including SMT	Cannot say	Patient A: 1 week and 3 years later Patient B: throughout care and 3 years later	No reported adverse consequences to occur with the intervention
Zhang, 2004 [35]	Children with acute otitis media < 2 mos, < 10 years old, no medical tx; *n* = 20	Low force (2–32 oz), Toftness chiropractic adjustment by a metered hand-held pressure applicator at the cervical, thoracic, lumbar and sacral contact site; number of adjustments range from 3 to 6	Cannot say	During the study period	No side effects or deterioration of clinical presentations were found to occur with the intervention
Paravicini, 2018 [66]	Male infants; 4.5–15 mos old; diagnosed with arthrogenic newborn torticollis, radiographs demonstrated rotational malposition and translation of atlas on axis in all cases; unresponsive to previous conservative tx methods; *n* = 6	Mobilization under anesthesia by doctor of chiropractic and assistant; atlas in full flexion; in cases of subluxated C1–2 articulation, a little traction was added; assistant chiropractor stabilized shoulders of sedated patient; line of drive along almost horizontal joint place with minimal force and no impulse	Cannot say	During the intervention	No AE occurred with the intervention
Alcantara, 2008 [47]	Patient A: 21-month-old; male; complaint of constipation since birth; Patient B: 7-month-old; female; complaint of constipation since 2 mos; Patient C: 21-month-old; female; encopresis and severe constipation since 10 months old; *n* = 3	Patient A: Decreased HVLA type thrusts; Activator technique, 3x/week for 3 weeks, 2x/week for 3 weeks, 1x/week (2 mos of care); dietary changes Patient B: 2x/week for 3 weeks; Activator Patient C: frequency not reported (2 mos of care); HVLA type thrust	Cannot say	Patient B: 1-yr FU, normal bowel movements Patient C: 3-yr FU normal bowel movements	Parents did not report any adverse reactions to occur with the intervention
Alcantara, 2010 [49]	Patient A: 7-year-old; male; ADHD; Adderall, Zoloft taken during chiropractic care; Patient B: 8-year-old; male; no medications; *n* = 2	Patient A: 20 visits over 32 weeks; proEFA supplement Patient B: 49 visits over 24 weeks HVLA type thrusts: diversified and Gonstead techniques	Cannot say	During the course of care	No AE documented/reported by patients or parents to occur with the intervention
Miller, 2008 [50]	Retrospective review of pediatric cases (patients = 781, total visits = 1310); < 3 years old; Chiropractic college teaching clinic; *n* = 781 (dismissed no treatment =82); 697 treated & reported outcomes; total visits = 5242	Patients receiving a type of chiropractic manipulation provided by interns (*n* = 697) Full spine pediatric SMT; *n* = 531 Occipital-sacral decompression; *n* = 50 Cervical spine pediatric SMT; *n* = 47 Thoracic spine pediatric SMT; *n* = 11 Lumbar spine pediatric SMT; *n* = 2 Pelvic pediatric SMT; *n* = 17 Other: *n* = 33	Negative side effects were detected by interpreting parental comments in the FU to the previous tx or same day as tx (*n* = 697). Defined as any adverse reaction reported by the parent. When an adverse reaction was reported by the parents, a description was detailed. Mild: transient and lasting < 24 h Moderate: requiring medical (general practitioner) tx Severe: requiring hospital tx	During the course of care	Male; 8 weeks old; post first cervical spine SMT tx for infant colic; parents called to report infant was not feeding well and was mildly distressed; following day parents report infant was fine and parents resumed care at the clinic Female; 8 weeks old; post 4th tx of cervical and thoracic SMT for infant colic; mother called to report infant had been crying since the tx; mother later reported the infant slept better than usual and resumed care at the clinic Female; 6 weeks old; few hours post first cervical spine SMT tx, parents reported a “head tilt”; infant was examined and presented with full range of motion and no antalgic posture; care continued Female; 7 weeks old; post first cervical spine SMT for infant colic; mother reported infant cried a lot, slept for 2 h, then awoke and continued to cry; continued for 3 more visits and self-discharged; at FU phone call mother reported infant was “doing fine” and did not require more care Male; 5 weeks old; FU with the parents; reported they would not attend the 7th visit because after the 6th visit of SMT for infant colic, the baby was restless and crying for almost 8 h; they did not continue with tx Male; 17 weeks old; reported birth trauma; on 25th visit immediately post pelvic SMT, infant began to crying, mother felt this was a cry of pain; a corrective ilium adjustment was performed by tutor and the baby stopped crying; mother called later that day to report child was fine; mother continued to bring her child for monitoring and care for next several months Female; 12 weeks old; on 11th visit, cervical spine SMT done for kinematic imbalance due to suboccipital strain, infant cried during tx and continued to cry after returning home; FU next day by phone, mother reported the infant was better but wished to stop tx

*AE* Adverse events, *D/t* Due to, *FU* Follow-up, *HA* Headache, *HVLA* high velocity low amplitude, *SMT* spinal manipulative therapy, *Months* mos, *RR* relative risk, *Tx* Treatment, *W/o* Without, *W/* With, *Wks* Weeks

^a^Results reported in this column cannot be used to infer about the risk of adverse events or the effectiveness of SMT

**Table 5:** Evidence Table: Case Reports

Author(s), Year	Subjects & Setting; n	Intervention(s)	Method of Measurement of AE	Follow-up	Key Findings^a^
Hubbard, 2010 [62]	7-year-old; female; migraine HA, mid-back and abdominal pain for previous 2 mos, episodic vomiting for intermittently for 9 mos	8-week course of low velocity, low-amplitude adjustments, following upper cervical pediatric protocol; 7 tx to C1 over 13 visits.	Cannot say	During course of tx	No report of adverse symptoms occurred after the intervention
Muir, 2012 [63]	5-year-old; male; ADHD (no medication): acting out, inability to follow instructions, poor home and school performance	11 tx over summer, 2-3x/week in November (re-evaluation at 4 wks, 2x/month between December–May); SMT, soft tissue therapy, and myofascial release therapy	Cannot say	1 year	No AE were reported
Bourque, 2018 [53]	5-month-old; male; fussing, irritability, crying, grunting, rigidity, abnormal position of left arm, 2 wks of constipation, breastfeeding difficulties on right side, apparent discomfort lying on stomach; fracture of left clavicle during birth	1x/week for 2 wks, 2 tx over 2 mos; sacro-occipital technic for occipital restriction, Thoracic spine (T2 and T5) was treated with the “touch and hold” technique by holding a specific, light pressure on the fixated vertebrae.	Cannot say	Patient A: 5 weeks Patient B: 4 weeks	No AE related with the intervention
Berube, 2004 [57]	6-day-old; female; symptoms of digestive disorder that began at 4-days-old, difficulty with eructation, taking several minutes to elicit, trouble eliminating stool accompanied by crying; immediate crying when lying supine	SMT performed with diversified technique modified for gestational age and size using low force; 1x/week, 4 weeks, re-evaluation with tx after 4 weeks	Cannot say	Cannot say	No AE due to chiropractic manipulation was reported by the parent
Dorough, 2018 [58]	2.5-year-old; male; speech delay, difficulty lying prone, unable to lift head up well, crying when pushing up from ground	Cervical spine modified Gonstead Technique and instrument-assisted Sigma-Instrument; 7 visits 1x/wk., 8 weeks	Cannot say	Over the course of treatment	No adverse reactions to tx were reported to occur with the intervention
Martin-Marcotte, 2018 [59]	21-month-old; female; episodes of constipation for the past 15 mos	Modified Diversified Technique for the child’s age and development; 2x/week, 4 weeks, re-evaluation after 10 visits, 1x/month subsequently	Cannot say	Over the course of treatment	No adverse reaction to adjustment reported
McCormick, 2018 [60]	15-month-old; male; motor developmental delay, not able to crawl, pull up to stand, stand alone or walk	Full spine SMT with Diversified Technique (Activator instrument-assisted); 1x/week for 4 weeks, 1x/every other week for 12 weeks	Cannot say	During the course of care	No adverse reactions were identified or reported to occur with the intervention
Lacroix, 2016 [64]	4-month-old; female; recurrent regurgitation after feeding, averse to being carried, difficult eructation, interrupted sleep, choking and rumination, wheezing during sleep, fussiness, distended stomach, excessive intestinal gas	17 chiropractic adjustments over 20 weeks; craniosacral technique and Diversified adjusting technique (high velocity low amplitude)	Cannot say	During the course of care	No AE were reported to occur with the intervention
Makela, 2018 [65]	3-year-old; female; autism spectrum disorder, no verbal or non-verbal communication, off balance when walking, toe-walking 50% of the time	SMT provided on 11 visits over 6 weeks; spring-loaded instrument assisted technique; after re-evaluation, 2x/week with re-evaluation every month (Dec – Mar)	Cannot say	During the course of care	No adverse reactions to treatment were reported
Dobson, 1996 [46]	5-year-old; male; asthmatic; seeks care to promote “normal” & vitality posture; ROM limited in extension; muscle tension cervical spine; neutral lateral radiograph revealed an os odontoideum	3x/week for 4 weeks, 4x/week for 2 weeks, 1x/week for 3 years; toggle-recoil (short lever high velocity, very low amplitude) adjustment when indicated	Cannot say	Cannot say	No negative effects were experienced with the intervention
Wilson, 2012 [48]	21-day-old; female; reported to pediatrician w/ concern of abnormality/ crepitus on back; presented to chiropractor due to fussiness and colic at 16-days-old	Day 23, follow-up investigation by child abuse center with the chiropractor confirmed the parents report. Parents described chiropractor initially held patient upside down by hips, with hands around hips and lower ribs. Applied pressure along spine with fingertips. Used a “spring-activated device” on back (in same location of fracture), while patient lay prone on the mother’s chest.	Chest radiograph and investigation by child abuse center to confirm reports	At 35 days of life, evidence of rib fracture healing with no new fractures	Acute fractures of 7th and 8th posterior ribs
Shafrir, 1992 [51]	4-month-old; male; head tilt noted in first week of life attributed to neck trauma during delivery, noted discomfort when placed on abdomen, could not raise head from prone; told would resolve but no improvement in head tilt after 4 months	First tx: Neck manipulation including flexion, extension and axial loading and unloading Second tx: parents returned after first response to manipulation, were reassured and infant was provided another neck manipulation	3 h post second tx, admitted to hospital; routine chest radiograph showed enlargement of the spinal canal from C3-T8. MRI of the head and spine showed a mass within the spinal cord, extending into the medulla superiorly and occupying the entire canal from mid-cervical to the lower thoracic region. During surgery, thrombosed veins were noted on the dorsum of the enlarged spinal cord, when spinal cord was incised at C6 level, creamy white, viscoelastic tumour tissue exuded spontaneously. No normal cord tissue was identifiable at this level. Cervical and lower thoracic portions of the tumour were easily removed from normal-appearing spinal cord tissue. Pathologic examination revealed mostly necrotic tissue, with the lack of inflammatory infiltrates (suggesting acute necrosis, rather than due to a high-grade malignancy), with several areas of low-grade astrocytoma.	Immediately after tx	After first tx: difficult to arouse him from a nap, he was described as limp, pale and moaning After second tx: immediately post-manipulation was alert, later began to moan and grunt continuously, fed poorly, fever developed. Three hours after second neck manipulation, he was admitted to the hospital, where he was described as listless and fussy, w/ a weak cry. Early next morning, he had a brief, generalized seizure, followed by “gasping” respirations and cyanosis, requiring tracheal intubation, followed by another 3 h seizure. Infant was admitted to the intensive care unit while comatose and rarely responsive to painful stimuli. Later, infant opened eyes and had conjugate movements. Infant had flaccid paralysis of both legs and right arm, with some active motion and withdrawal of the left arm. Post-operatively, infant regained motor and sensory function to the T4 level. 18 months later, he had full use of the upper extremities, sensory function at approximately T9 level and some spontaneous but non-functional motion of the right leg. Diagnosis: congenital spinal cord astrocytoma
Humphris, 2014 [52]	6-month-old; female; left head rotation and ipsilateral flattening of her posterolateral cranium, frequent regurgitation of breast milk immediately after feeding with inability to feed from the right breast, unsettled sleep patterns	3 visits over 4 months; Diversified technique with a light, modified, HVLA impulse; no other interventions provided	Cannot say	Cannot say	No AE were reported or observed to occur with the intervention
Fairest, 2013 [54]	6-week-old; female; left-sided cranial flattening and favored left head rotation, occasional regurgitation of an entire breastfeed immediately after feeding, groaning when placed prone in an inclined position, unsettled sleep patterns; advised by GP & midwife to seek chiropractic care	1x/week, 10 weeks; 7 visits included Diversified technique (modified HVLA thrust) to cervical (7 visits) and sacrum (1 visit) and Activator to thoracic (2 visits), 3 visits of no SMT	Cannot say	Cannot say	No AE were observed, nor reported to occur with the intervention
Gordon, 2011 [55]	2-week-old; male; facial and upper limb postural asymmetry following a forceps-assisted vaginal birth after Caesarean, droopy lip on the right, right arm assumed waiter’s tip posture at rest	Chiropractic craniosacral techniques: low-force static hold adjustments to cervical and sacral segments; soft tissue therapy to cervical muscles; 2x/week for 2 weeks, then 1x/every other week for 12 weeks	Cannot say	Cannot say	No adverse effects of management were reported

*AE* Adverse events, *D/t* Due to, *FU* Follow-up, *HA* Headache, *HVLA* high velocity low amplitude, *SMT* spinal manipulative therapy, *Months* mos, *RR* relative risk, *Tx* Treatment, *W/o* Without, *W/* With, *Wks* Weeks

^a^Results reported in this column cannot be used to infer about the risk of adverse events or the effectiveness of SMT

### Statistical analyses

When data were available, we computed the incidence (and 95% confidence intervals) of adverse events and relative risk (and 95% confidence intervals) from RCTs and cohort studies. Incidence was measured by calculating the number of adverse events in a group divided by the total number of participants in the same group [36]. Relative risk was measured by dividing the incidence of adverse events in the intervention group by the incidence of adverse events in the comparison group [36]. Confidence intervals (CI) were calculated using incidence of adverse events in each group, total number of participants in each group, and α = 0.05.

### Evidence synthesis

We used best evidence synthesis methodology to synthesize evidence from high and acceptable quality studies [37]. The evidence synthesis provides conclusions based on the best available evidence or may conclude that there is insufficient evidence to make any conclusions [37].

### Reporting of outcomes

For the two RCTs included in our review, we checked the clinical trials registry for evidence of selective reporting of outcomes or protocol changes. Sawyer et al. was not found in the registry, as it was published in 1999. We retrieved the protocol by Miller et al. (#NCT01513304) which listed a daily crying diary as the primary outcome (no further information about secondary outcomes was provided).

## Results

### Study selection

**Fig. 1:**
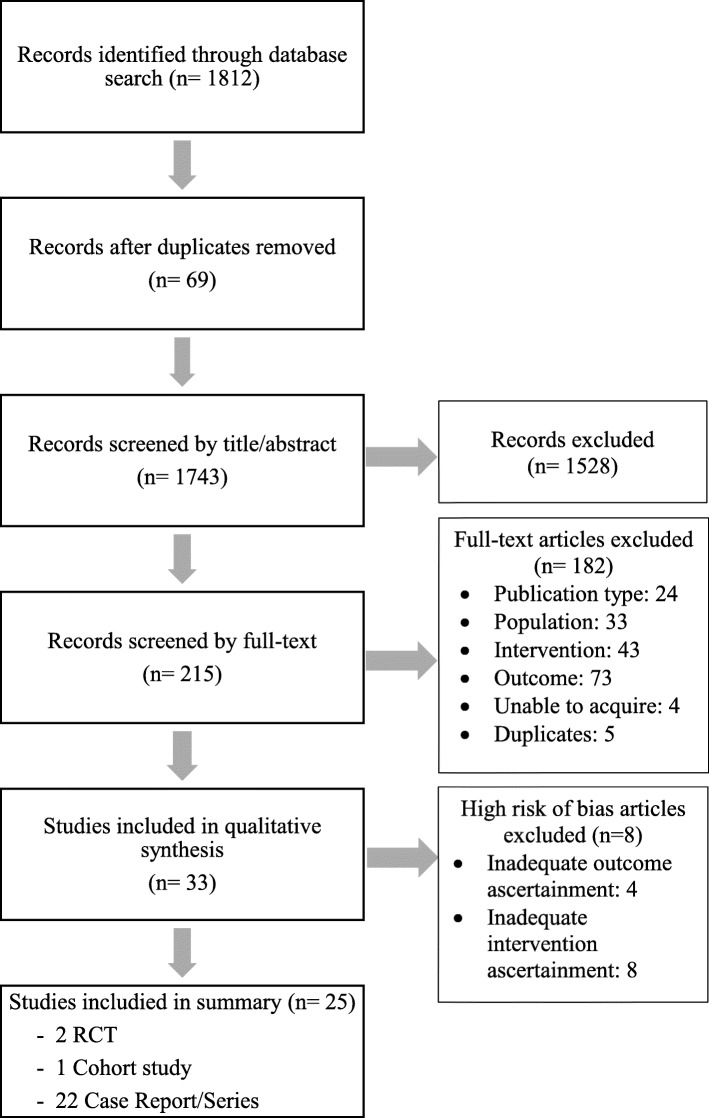
Flow diagram of study selection

Our search retrieved 1812 citations (Fig. 1). We removed 69 duplicates and screened 1743 articles. Interrater agreement for phase 1 screening was 95.4% between MC and PC. We screened 215 full-text articles (phase 2). Of those, 33 articles met the inclusion criteria and were eligible for critical appraisal. Reasons for exclusion during phase 2 screening were ineligible publication type (*n* = 24), population ≥ 10 years old (*n* = 33), intervention did not include SMT (*n* = 43), and outcomes did not include adverse events (*n* = 73).

### Risk of Bias within studies

We critically appraised 33 studies. Of those, eight had a low or unacceptable quality and were excluded from the evidence synthesis [38–45]. These included seven case reports or case series, and one cohort study. High risk of bias studies had the following methodological limitations: 1) inadequate outcome ascertainment (*n* = 4) and 2) inadequate exposure ascertainment (*n* = 8) (Tables 6, 7 and 8). Twenty-five studies with high or acceptable quality were included in our evidence synthesis [32–35, 46–66]. Nevertheless, these studies had some methodological limitations including inadequate reporting of adverse events measurement (Tables 6, 7 and 8). We contacted authors from two studies to inquire about the measurements of adverse events [32, 33]. Sawyer et al. clarified that events were measured from reports by the chiropractor at each visit. Similarly, Saedt et al. indicated that adverse events were reported by the manual therapist after each treatment, and further defined the various vegetative responses reported as adverse events. For example, hyperextension (of the trunk or cervical spine) is a reaction an infant can make when they feel discomfort and perspiration is a slight sweating reaction of the infant to the intervention.

**Table 6:** Risk of Bias table: Low risk of bias - RCT

Author, Year	1.1	1.2	1.3	1.4	1.5	1.6	1.7	1.8	1.9	1.10	Overall assessment
Miller, 2012 [61]	Y	Y	Y	Y	Y	Y	CS	Tx blinded: 0 Tx not-blinded: 0 No tx: 12	Y	N/A	+
Sawyer, 1999 [32]	Y	Y	Y	Y	N	Y	CS	SMT: 0% (0/9) Placebo: 9% (1/11)	Y	N/A	+

*Y* yes, *N* no, *N/A* not applicable, *CS* can’t say; ++: high quality; +: acceptable quality; −: unacceptable quality/rejected

Legend: RCTs

1.1 Research Question

1.2 Method of Randomization

1.3 Concealment

1.4 Blinding

1.5 Baseline Characteristics

1. 6 Contamination

1.7 Outcome Measurement

1.8 Lost to Follow-Up

1.9 Intention-to-Treat

1.10 Between Sites

**Table 7:** Risk of Bias table: Cohort Studies

**Risk of Bias table: Low risk of bias - Cohort Studies**															
**Author, Year**	**1.1**	**1.2**	**1.3**	**1.4**	**1.5**	**1.6**	**1.7**	**1.8**	**1.9**	**1.10**	**1.11**	**1.12**	**1.13**	**1.14**	**Overall Assessment**
Saedt, 2018 [33]	Y	N/A	Y	N/A	CS	N/A	Y	N/A	CS	CS	N	N/A	N/A	N	+
**Risk of Bias table: High risk of bias - Cohort Studies**															
**Author, Year**	**1.1**	**1.2**	**1.3**	**1.4**	**1.5**	**1.6**	**1.7**	**1.8**	**1.9**	**1.10**	**1.11**	**1.12**	**1.13**	**1.14**	**Overall Assessment**
Douglas, 2016 [74]	Y	N/A	N	N/A	0%	N/A	CS	N/A	N	CS	N	N/A	N/A	Y	–

*Y* yes, *N* no, *N/A* not applicable, *CS* can’t say; ++: high quality; +: acceptable quality; −: unacceptable quality/rejected

Legend: Cohort Studies

1.1 Research Question

1.2 Comparable Sources

1.3 % Participation

1.4 Outcome Analysis

1.5 % Drop-out

1.6 Compare Loss to Follow-Up

1.7 Outcome Defined

1.8 Blinding

1.9 Acknowledge Bias

1.10 Reliability of Exposure

1.11 Other Sources for Reliability

1.12 Measurements Occur > 1 time

1.13 Confounders

1.14 Confidence Intervals

**Table 8:** Risk of Bias table: Case Report & Case Series

**Risk of Bias table: Low risk of bias - Case Report & Case Series**									
**Author, Year**	**1.1**	**1.2**	**1.3**	**1.4**	**1.5**	**1.6**	**1.7**	**1.8**	**Overall assessment**
Hubbard, 2010 [62]	CS	Y	CS	N	N/A	CS	Y	Y	+
Muir, 2012 [63]	CS	Y	CS	Y	N/A	CS	Y	Y	+
Bourque, 2018 [53]	CS	Y	CS	N	N/A	CS	CS	Y	+
Iyer, 2017 [34]	CS	Y	CS	Y	N/A	CS	CS	Y	+
Young, 2017 [56]	CS	Y	CS	CS	N/A	CS	CS	Y	+
Berube, 2004 [57]	CS	Y	CS	Y	N/A	CS	CS	Y	+
Dorough, 2018 [58]	CS	Y	CS	N	N/A	CS	CS	Y	+
Zhang, 2004 [35]	Y	Y	CS	CS	N/A	CS	CS	N	+
Martin-Marcotte, 2018 [59]	CS	Y	CS	CS	N/A	CS	CS	Y	+
McCormick, 2018 [60]	CS	Y	CS	CS	N/A	CS	CS	Y	+
Lacroix, 2016 [64]	CS	Y	CS	CS	N/A	CS	CS	Y	+
Makela, 2018 [65]	CS	Y	CS	CS	N/A	CS	Y	Y	+
Paravicini, 2018 [66]	Y	Y	CS	CS	N/A	CS	Y	Y	+
Dobson, 1996 [46]	CS	Y	CS	CS	N/A	CS	CS	Y	+
Alcantara, 2008 [47]	CS	Y	CS	CS	N/A	CS	Y	Y	+
Wilson, 2012 [48]	CS	Y	Y	Y	N/A	CS	Y	Y	+
Alcantara, 2010 [49]	Y	Y	CS	CS	N/A	CS	CS	Y	+
Miller, 2008 [50]	Y	Y	Y	Y	N/A	CS	Y	Y	+
Shafrir, 1992 [51]	CS	Y	Y	Y	N/A	CS	Y	Y	+
Humphris, 2014 [52]	CS	Y	CS	Y	N/A	CS	CS	Y	+
Fairest, 2013 [54]	CS	Y	CS	Y	N/A	CS	CS	Y	+
Gordon, 2011 [55]	CS	Y	CS	CS	N/A	CS	CS	Y	+
**Risk of Bias table: High risk of bias - Case Report & Case Series**									
**Author, Year**	**1.1**	**1.2**	**1.3**	**1.4**	**1.5**	**1.6**	**1.7**	**1.8**	**Overall assessment**
Kinkpe, 2009 [39]	CS	CS	Y	N	N/A	CS	Y	N	–
Nicolas-Schmid, 2016 [40]	Y	N	Y	CS	N/A	CS	N	N	–
Cox, 2016 [41]	Y	N	CS	CS	N/A	CS	CS	Y	–
Miller, 2009 [42]	Y	CS	CS	CS	N/A	CS	CS	Y	–
Ghanim, 2019 [43]	CS	N	Y	Y	N/A	CS	Y	Y	–
Deputy, 2014 [44]	CS	CS	Y	N	N/A	CS	Y	Y	–
Wiberg, 2010 [45]	Y	CS	CS	CS	N/A	CS	CS	N	–

*Y* yes, *N* no, *N/A* not applicable, *CS* can’t say; ++: high quality; +: acceptable quality; −: unacceptable quality/rejected

Legend: Case report & case series

1.1 Patient Selection

1.2 Exposure Ascertainment

1.3 Outcome Ascertainment

1.4 Alternative Causes

1.5 Challenge/Rechallenge

1.6 Dose-Response

1.7 Length of Follow-Up

1.8 Sufficient Detail

### Study characteristics

We included two RCTs, [32, 61] one cohort study [33] and 22 case reports/series [34, 35, 46–60, 62–66] (Tables 2, 3, 4 and 5). All spinal manipulations and mobilizations were provided by chiropractors. One RCT tested the effectiveness of spinal mobilization for the management of infants with unexplained persistent crying [61]. The second RCT investigated the effectiveness of high-velocity, low-amplitude SMT for the management of children with acute otitis media [32]. In the cohort study, spinal mobilization was used to treat infants with upper cervical dysfunction [33]. In five case reports or case series the intervention was spinal mobilization, [34, 53, 55, 62, 66] seven provided instrument-assisted SMT, [35, 47, 48, 56, 58, 60, 65] and ten provided high-velocity, low-amplitude SMT modified for the age and development of the patient [46, 49–52, 54, 57, 59, 63, 64].

#### Description of adverse events

Adverse events were described in five studies; with one study describing a severe adverse event (case report), one describing an indirect harm (case report), and three studies describing mild adverse events (one RCT, one cohort study, one case series).

Regarding the severe adverse event, acute fractures of the posterior 7th and 8th ribs occurred in a 21-day-old female treated for fussiness and colic, after the use of a spring-activated device on the infant’s back [48]. An indirect harm occurred in a 4-month-old male who presented to a chiropractor with head tilt. He was treated with spinal manipulation and a diagnosis of congenital spinal cord astrocytoma was delayed. After the second visit, the child was hospitalized and parents reported the child was difficult to arouse from sleep, limp, pallor, moaning, poor feeding and fever [51].

Three studies described mild adverse events. In an RCT of children between the ages of 6 months and 6 years with acute otitis media, one parent reported mid-back soreness and one reported irritability during a course of 10 treatments of high-velocity, low-amplitude SMT to the cervical region [32]. In a cohort study of infants less than 27 weeks old with indications of upper cervical dysfunction, adverse events reported by the chiropractor included vegetative responses such as flushing, hyper-extension, perspiration, and gastro-esophageal reflux, and short breathing pattern changes after mild mobilization techniques focusing on C1 [33]. In a case series, four male and female 5 to 8-week-old infants presenting with colic were treated with cervical and thoracic pediatric SMT. Parents reported adverse events of poor feeding, mild distress, and increased crying [50]. The parent of a 6-week-old female infant reported a head tilt after cervical SMT [50]. A 17-week-old male infant started to cry immediately after pelvic SMT which resolved after a corrective SMT, with no residual complaints [50]. The mother of a 12-week-old female infant reported crying during and following a cervical spine SMT treatment for suboccipital strain [50].

No adverse events associated with SMT were reported in one RCT, [61] six case series [34, 35, 47, 49, 56, 66] and 13 case reports [46, 52–55, 57–60, 62–65].

#### Incidence of adverse events

We computed the incidence of adverse events associated with SMT using data from two RCTs and one cohort study (Tables 2, 3, 4 and 5). In a RCT of infants less than 8 weeks old treated for unexplained persistent crying, the incidence of increased crying in the low force SMT group was 0% compared to 2.94% (95% CI: 0.52, 14.92) in the no SMT group [61]. In another RCT of children between the ages of 6 months and 6 years old with otitis media, the incidence of mild adverse events (mid-back soreness and irritability) was 22.22% (95% CI: 6.32, 54.74) after full spine high-velocity, low-amplitude SMT (focusing on the cervical region) compared to 9.09% (95% CI: 1.62, 37.74) in the placebo group (increased crying) [32]. In a cohort study of infants less than 27 weeks old treated for upper cervical dysfunction with mild mobilization techniques of C1, the incidence of clinician recorded mild adverse events ranged from 0.3% (gastro-esophageal reflux) to 17.8% (flushing) [33].

#### Association between SMT and adverse events

In one small RCT of children with acute otitis media treated with high-velocity, low-amplitude SMT primarily to the cervical region (n_SMT_ = 9; n_placebo_ = 11), the relative risk of mild adverse events associated with SMT compared with placebo was 2.44 (95% CI: 0.26, 22.8) [32].

## Discussion

A limited body of evidence of adequate methodological quality is available to describe and quantify the risk of adverse events associated with SMT in children under 10 years of age. Although serious adverse events are reported in the literature, the risk of serious adverse events remains unknown in this population [48, 51]. Most studies report mild and transient adverse events (e.g., increased crying, soreness, irritability). Our review suggests that the risk of mild adverse events ranges from 0.3% (95% CI: 0.06, 1.82) in infants < 27 weeks old treated with mild mobilization of C1 for upper cervical dysfunction to 22.22% (95% CI: 6.32, 54.74) in children aged between 6 months and 6 years treated for otitis media. We observe that the methods used to measure adverse events are of questionable validity and reliability.

Our review improved on the methodology of previous reviews by evaluating the internal validity of case reports and case series. This is important because the quality of these designs is highly variable and therefore even the description of an observation can be biased [67, 68]. In comparison to Vohra et al. [13], our study collected one additional case report published in 2012, which reported rib fractures in a 21-day-old infant [48]; this study was included in other reviews [15, 18]. Our review did not include 11 of the 13 studies included in the review by Vohra et al. [13]; one case study was in German, four studies (one RCT, one case report and two case series) did not differentiate a population less than 10 years old, and six studies (four case reports and two case series) were published prior to 1990. In comparison to Todd et al. [15], our review did not include 28 of the 31 studies included in their review. One case report was not in English, seven studies (two RCTs, one cohort study, three case series and one case report) did not distinguish between or include participants less than 10 years old, eight studies (two RCTs, four case series and one case report) did not provide SMT or were unclear whether every participant received SMT, and 12 were systematic or narrative reviews. A number of adverse events in this review were summarized from other systematic or narrative reviews and not directly from the original report in the literature. In comparison to Humphreys et al., [18] our review did not include three case series because one case series did not distinguish participants under the age of 10 years and two case series did not make it clear that the participants were receiving SMT. None of the previously published reviews included the RCT by Miller et al. (2012), which reported no adverse events in the SMT group and one mild adverse event in the comparison group, [61] nor the cohort study by Saedt et al. (2018) that reported multiple mild adverse events [33]. None of these reviews appraised the methodological quality of studies included in their evidence synthesis. Finally, our review improves on the quality of the other systematic reviews because we computed incidence rates and relative risks where possible.

Methodological differences between previous reviews and our review are important to note because variations in methodology can lead to different conclusions [69, 70]. In their review, Vohra et al. report nine serious adverse events and 20 cases of delayed diagnosis associated with SMT [13]. Similarly, the review by Todd et al. reported 15 serious adverse events and 775 mild to moderate adverse events following manual therapy [15]. Our results differ from the findings of those reviews because we excluded studies of poor methodological quality, and those where the use of SMT was unclear [13, 15]. Moreover, our review is up to date and includes recently published studies. We improved on the methodology of previous reviews [13, 15, 18] by excluding case reports where the exposure (i.e. SMT) and outcome (i.e. adverse event) were not adequately measured. Serious and severe adverse events following SMT may be inappropriately reported in the literature [13, 15] because most included studies that were not designed nor powered to measure these rare events. Therefore, we recommend future epidemiological studies be designed to specifically estimate the incidence of adverse events within well-defined populations of individuals treated with SMT.

Strengths of our study include adhering to PRISMA and PRISMA harms reporting checklists (Additional file 3), [22, 23] a protocol established prior to completion and registration with PROSPERO, a clear research question, a robust literature search strategy reviewed by two librarians, screening inter-rater reliability comparison, critical appraisal of eligible studies and a review process by senior scientists at each step of the rapid review. We also included all original research study types (RCTs, cohort studies, case-control studies, case series and case reports) to adequately inform each of our research aims and provided a full electronic search strategy for at least one database including limits used, so our search could be repeated [23]. Our study has limitations. We may have missed studies in our search. A recent study demonstrated that searching MEDLINE alone captured 92% of papers per systematic review regarding adverse effects of surgical interventions but only 65% of those for non-surgical similar effects [71]. The authors recommended that for non-surgical interventions, the search filter should include specific and generic adverse events terms and clearly specify the intervention. Consistent with this recommendation, our search filter included specific terms related to adverse events and harms, as well as SMT related terms. We applied this filter to three databases that capture the majority of manual therapy studies, and that also complies with the WHO rapid review methodology guidelines. We only included studies published in English; however, the majority of studies are published in English [72]. Finally, we only included participants less than 10 years old. While this was specific to our research question, many studies do not categorize participants based on age, which led to a large number of studies being excluded from our review.

On September 30th, 2019, the College of Chiropractors of British Columbia released a public notice reporting on the results of the board regarding SMT treatment of children under the age of 10 years [73]. They determined that the treatment of children with SMT does not pose a significant risk to the public and the College of Chiropractors of British Columbia is not pursuing regulatory action at this time [73]. The results of our rapid review were not the only documents reviewed in their analysis. We did not assess the efficacy or effectiveness of SMT for the management of children under the age of 10 years. Therefore, the results of our review cannot be used to make inferences about the risk-benefit ratio of SMT in this population. We recommend that the future development of public and regulatory policies about the use of SMT to treat children include a review of the literature on the efficacy and/or effectiveness of SMT.

Very little is known about the risk of severe and serious adverse events related to SMT in children below the age of 10 years. In one of the few population-based active surveillance studies involving 2500 Canadian pediatricians and pediatric subspecialties, providing coverage to about 7 million children less than 18 years of age, 12 cases of serious adverse events were reported over a two-year period. Of these, eight were adjudicated for risks associated with complementary and alternative medicine use, of which one was possibly related to SMT provided by a chiropractor in a 13-year-old patient. No serious adverse events involving SMT in patients less than 10 years of age were reported [14]. This suggests that these severe and serious adverse events are rare in the general population and studying this issue would require a large sample size. We recommend the implementation of a population-based active surveillance program to measure the incidence of severe and serious adverse events following SMT treatment in this population. Research is needed to determine the incidence of adverse events associated with SMT in children. Moreover, future research must improve on the clarity and definition of SMT and use a standardized and valid tool to measure adverse events in children [14]. Epidemiological studies are also urgently needed to determine whether SMT is associated with an increased risk of severe and serious adverse events. Due to their rarity, it is unlikely that a large enough number of severe and serious adverse events can be captured in RCTs designed to determine the effectiveness of SMT. Therefore, we recommend the design and conduct of population-based case-control (including case-crossover studies), or cohort studies to measure the association between SMT and severe or serious adverse events. It is likely that this type of research can only be done using high-quality population-based administrative databases.

## Conclusion

Most studies report mild and transient adverse events (e.g., increased crying, soreness, irritability) in children under 10 years old receiving SMT. The risk of moderate and severe adverse events is unknown in children treated with SMT. It is unclear whether SMT increases the risk of adverse events in children < 10 years old. Research is urgently needed to determine the incidence of adverse events associated with SMT in children.

## Supplementary information

Additional file 1: **Appendix 1.** MEDLINE Search Strategy.

Additional file 2: **Appendix 2:** Methodology Checklist: Case Report & Case Series.

Additional file 3: PRISMA.

## Data Availability

The datasets used and/or analyzed during the current study are available from the corresponding author on reasonable request.

## Abbreviations

CI: Confidence intervals
MSK: Musculoskeletal
RCT: Randomized controlled trial
SIGN: Scottish Intercollegiate Guidelines Network
SMT: Spinal manipulative therapy
