# Supplementary material for: The safety of spinal manipulative therapy in children under 10 years: a rapid review
Source: Chiropr Man Therap. 2020 Feb 25;28:12. doi: 10.1186/s12998-020-0299-y (PMC7041232; doi:10.1186/s12998-020-0299-y)
Supplement: Supplementary file 1 — Appendix 1. MEDLINE Search Strategy. [file 12998_2020_299_MOESM1_ESM.docx]

**APPENDIX 1: MEDLINE Search Strategy**

Search run August 1, 2019 in Ovid MEDLINE: Epub Ahead of Print, In-Process & Other Non-Indexed Citations, Ovid MEDLINE® Daily and Ovid MEDLINE® 1946-Present; 1485 results

| 1 | exp Infant/ | 1102731 |
| --- | --- | --- |
| 2 | Child, Preschool/ | 885237 |
| 3 | Child/ | 1626074 |
| 4 | Pediatrics/ | 51489 |
| 5 | (baby or babies).ab,kf,ti. | 67527 |
| 6 | "newborn*".ab,kf,ti. | 169701 |
| 7 | (infant or infants).ab,kf,ti. | 397533 |
| 8 | (child or children*).ab,kf,ti. | 1214666 |
| 9 | (pediatric* or paediatric*).ab,kf,ti. | 338046 |
| 10 | (young adj3 (person* or people)).ab,kf,ti. | 29867 |
| 11 | or/1-10 [**pediatric population] | 2896830 |
| 12 | Musculoskeletal Manipulations/ | 1621 |
| 13 | Manipulation, Spinal/ | 1507 |
| 14 | Manipulation, Chiropractic/ | 949 |
| 15 | Manipulation, Orthopedic/ | 3834 |
| 16 | Manipulation, Osteopathic/ | 980 |
| 17 | (Activator adj (method or tool*)).ab,kf,ti. | 7 |
| 18 | (adjust* adj3 (chiropract* or spinal or lumbar or cervical or thoracic or instrument* or tool* or electric)).ab,kf,ti. | 1659 |
| 19 | (flexion-distraction or flexion distraction).ab,kf,ti. | 255 |
| 20 | (HVLA or high velocity low amplitude).ab,kf,ti. | 256 |
| 21 | (manipulat* adj3 (chiropract* or naprapath* or osteopath* or orthopedic* or orthopaedic*)).ab,kf,ti. | 1639 |
| 22 | (manipulat* adj3 (spinal or spine or low* back or joint* or lumbar or neck or thoracic or cervical or MSK or musculoskeletal or vertebr*)).ab,kf,ti. | 3506 |
| 23 | (manipulat* adj3 (instrument assisted or instrument-assisted)).ab,kf,ti. | 8 |
| 24 | (manipulat* adj3 (physiotherap* or physical therap*)).ab,kf,ti. | 256 |
| 25 | (mobili?at* adj3 (chiropract* or naprapath* or osteopath* or orthopedic* or orthopaedic*)).ab,kf,ti. | 29 |
| 26 | (mobili?at* adj3 (spinal or spine or low* back or joint* or lumbar or neck or thoracic or cervical or MSK or musculoskeletal or vertebr*)).ab,kf,ti. | 1198 |
| 27 | ((manipulat* or mobili?at*) adj4 instrument*).ab,kf,ti. | 736 |
| 28 | ((therap* or treat* or intervention* or manag*) adj3 (manual or manipulat* or mobili?at* or MSK or musculoskeletal)).ab,kf,ti. | 16046 |
| 29 | or/12-28 [**intervention] | 26578 |
| 30 | Case-Control Studies/ | 268088 |
| 31 | case reports.pt. | 2035015 |
| 32 | Cohort Studies/ | 243338 |
| 33 | controlled clinical trial.pt. | 93185 |
| 34 | Controlled Clinical Trials as Topic/ | 5439 |
| 35 | Cross-Over Studies/ | 45653 |
| 36 | Double Blind Method/ | 152412 |
| 37 | Epidemiologic Studies/ | 8035 |
| 38 | Follow-Up Studies/ | 618433 |
| 39 | Longitudinal Studies/ | 125240 |
| 40 | Observational Study.pt. | 64796 |
| 41 | Prospective Studies/ | 508866 |
| 42 | Random Allocation/ | 99813 |
| 43 | randomized controlled trial.pt. | 486353 |
| 44 | exp Randomized Controlled Trial/ | 486942 |
| 45 | Randomized Controlled Trials as Topic/ | 125430 |
| 46 | Retrospective Studies/ | 760923 |
| 47 | Single Blind Method/ | 27104 |
| 48 | (case adj3 (control* or series or report*)).ab,kf,ti. | 751415 |
| 49 | ((crossover or cross-over) adj3 (study or studies or trial*)).ab,kf,ti. | 42929 |
| 50 | ((followup or follow-up) adj3 (stud* or design* or analysis)).ab,kf,ti. | 77680 |
| 51 | "cohort*".ab,kf,ti. | 528994 |
| 52 | (control* adj3 trial*).ab,kf,ti. | 262682 |
| 53 | (longitudinal* adj3 (stud* or design* or analysis)).ab,kf,ti. | 111302 |
| 54 | (prospective adj3 (stud* or design* or analysis)).ab,kf,ti. | 359480 |
| 55 | (random* adj5 (allocat* or assign* or control* or clinical or patient* or sample* or trial*)).ab,kf,ti. | 622915 |
| 56 | (retrospective adj3 (stud* or design*)).ab,kf,ti. | 276929 |
| 57 | ((singl* or doubl* or treb* or tripl*) adj3 (blind* or mask*)).ab,kf,ti. | 166868 |
| 58 | or/30-57 [**study designs] | 5472722 |
| 59 | Patient Harm/ | 135 |
| 60 | Patient Safety/ | 17305 |
| 61 | Risk/ | 119194 |
| 62 | Risk Assessment/ | 245171 |
| 63 | Risk Factors/ | 776722 |
| 64 | adverse effects.fs. | 1661662 |
| 65 | (adverse adj3 (effect* or event* or reaction*)).ab,kf,ti. | 362549 |
| 66 | complication*.ab,kf,ti. | 923202 |
| 67 | "harm*".ab,kf,ti. | 166972 |
| 68 | "risk*".ab,kf,ti. | 2062390 |
| 69 | (safe or safety).ab,kf,ti. | 723522 |
| 70 | or/59-69 [**adverse effects] | 4856198 |
| 71 | 11 and 29 and 58 | 1529 |
| 72 | 11 and 29 and 70 | 1014 |
| 73 | 71 or 72 | 1938 |
| 74 | limit 73 to (english language and yr="1990 - 2020") | 1544 |
| 75 | limit 74 to (address or clinical conference or comment or congress or consensus development conference or consensus development conference, nih or editorial or government document or guideline or letter or meta analysis or news or practice guideline) | 59 |
| 76 | 74 not 75 | 1485 |
