# Supplementary material for: The safety of spinal manipulative therapy in children under 10 years: a rapid review
Source: Chiropr Man Therap. 2020 Feb 25;28:12. doi: 10.1186/s12998-020-0299-y (PMC7041232; doi:10.1186/s12998-020-0299-y)
Supplement: Supplementary file 2 — Appendix 2: Methodology Checklist: Case Report & Case Series. [file 12998_2020_299_MOESM2_ESM.docx]

**APPENDIX 2: Methodology Checklist: Case Report & Case Series**

Adapted from Murad et al. Methodological quality and synthesis of case series and case reports. *BMJ Evidence-Based Medicine*. 2018; 23:60-63.

| Study identification *(Include author, title, year of publication, journal title, pages)* | | | | | |
| --- | --- | --- | --- | --- | --- |
| Guideline topic: | | Key Question No: | | Reviewer: | |
| Before completing this checklist, consider:   1. Is the paper really a case report or case series? 2. Is the paper relevant to key question? Analyze using PICO. IF NO, REJECT (give reason below). IF YES, complete the checklist. | | | | | |
| Reason for rejection:   - Paper not relevant to key question - Other reason: | | | | | |
| SECTION 1: INTERNAL VALIDITY | | | | | |
| SELECTION | | | | | |
| 1.1 | Does the patient(s) represent(s) the whole experience of the investigator (centre) or is the selection method unclear to the extent that other patients with similar presentation may have not been reported?  The author should provide a rationale for publication of this patient case, and not others within their medical centre. There might be multiple cases that have presented to the investigator/medical centre over a certain period of time, which should be reported or referred to. In contrast, a study might be unclear as to how the patient case was selected, questioning whether the report reflects the whole experience of the investigator/centre or was subject to selection bias. | | - Yes - Can’t say | | - No |
| ASCERTAINMENT | | | | | |
| 1.2 | Was the exposure adequately ascertained?  The case report must describe the method of determining exposure. Was the method valid and reliable? Do they provide references for validity and reliability? For example, self-report is less reliable than administrative or billing codes, which in turn would be less reliable than clinical records. | | - Yes - Can’t say | | - No |
| 1.3 | Was the outcome adequately ascertained?  The case report must describe the method of determining the outcome. Were the methods valid and reliable? Do they provide references for validity and reliability? If not, are there validated and reliable questionnaires available for use? For example, self-report is less reliable than administrative or billing codes, which in turn would be less reliable than clinical records. | | - Yes - Can’t say | | - No |
| CAUSALITY | | | | | |
| 1.4 | Were other alternative causes that may explain the observation ruled out?  Further questions or investigations should be performed to determine the use of other or concurrent interventions or contributions that may have an effect on the outcome of the case. | | - Yes - Can’t say | | - No |
| 1.5 | Was there a challenge/rechallenge phenomenon?  Stronger inferences in a case report can be made if the drug/intervention was administered, removed, and subsequently administered again with reciprocal changes in the outcome. For example, if an adverse drug reaction resolved with cessation of the drug and reoccurred after reintroduction of the drug. | | - Yes - Can’t say | | - No - N/A |
| 1.6 | Was there a dose-response effect?  The author should report on intervention or drug doses and the response to each of these, to determine whether there are differential clinical outcomes or adverse reactions. Stronger inferences can be made if responses vary in intensity based on doses provided. Important information may be garnered regarding minimal effective dose, maximum effective dose/plateau of intervention outcomes, or adverse reactions. | | - Yes - Can’t say | | - No - N/A |
| 1.7 | Was follow-up long enough for outcomes to occur?  The follow-up period must be sufficient in length to determine whether the intervention has reached its full effect or whether adverse reactions are associated with its use. A follow-up period that is insufficient may not adequately report the effects or side-effects of the intervention. | | - Yes - Can’t say | | - No |
| REPORTING | | | | | |
| 1.8 | Is the case(s) described with sufficient details to allow other investigators to replicate the research or to allow practitioners make inferences related to their own practice?  A case report that is described with sufficient details may allow readers to apply the evidence derived from the report in their practice. Details should include the clinical course of the condition, exposures, symptoms, signs, interventions and outcomes. If inadequately reported, will likely be unhelpful in the course of clinical care. | | - Yes | | - No |
| *Questions 1.4, 1.5 and 1.6 are mostly relevant to cases of adverse events. | | | | | |

| SECTION 2: OVERALL ASSESSMENT OF THE STUDY | | | |
| --- | --- | --- | --- |
| 2.1 | Overall judgement about methodological quality based on the questions deemed most critical in the specific clinical scenario: | - Acceptable - Unacceptable/Reject | |
| 2.2 | Are the results of this study directly applicable to the patient group targeted by this review? | - Yes | - No |
| 2.3 | Notes: summarize the authors conclusions. Add any comments on your own assessment of the study, and the extent to which it answers your question and mention any areas of uncertainty raised above. | | |
|  |  | | |

**Glossary**

Case report: Description of the clinical course of one individual, which may include particular exposures, symptoms, signs, interventions or outcomes. A case report is the smallest publishable unit in the literature.

Case series: Description of the clinical course of one individual, which may include particular exposures, symptoms, signs, interventions or outcomes. Case series aggregate of individual cases in one publication.

Adverse events: Any unfavorable sign, symptom, or disease temporally associated with the treatment, whether or not caused by the treatment.
