# Supplementary figures and images for: The safety of spinal manipulative therapy in children under 10 years: a rapid review
Source: Chiropr Man Therap. 2020 Feb 25;28:12. doi: 10.1186/s12998-020-0299-y (PMC7041232; doi:10.1186/s12998-020-0299-y)

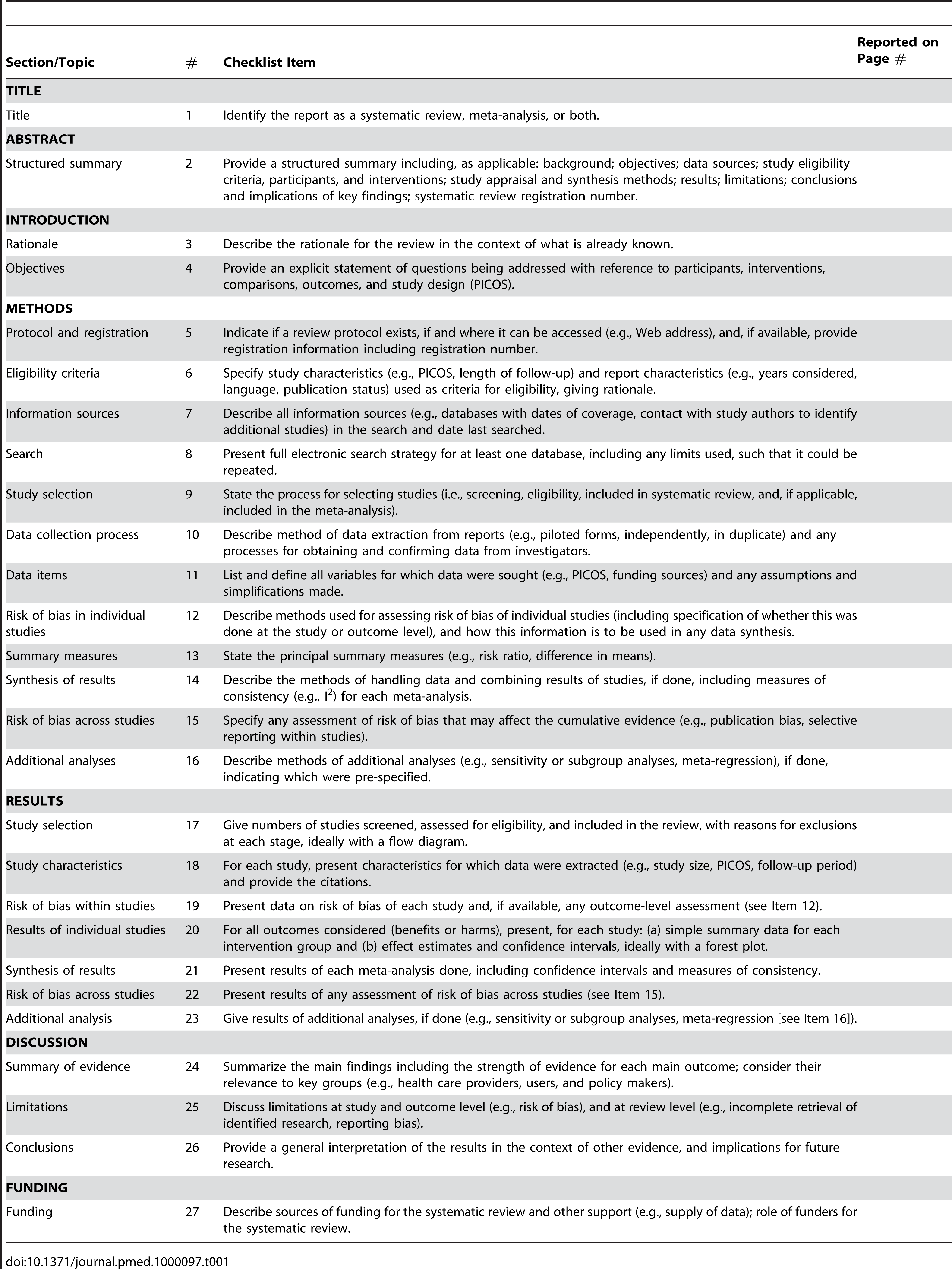

Supplement: Supplementary file 3 — PRISMA. [file 12998_2020_299_MOESM3_ESM.png]
